# Dataset on the imprint of the Agia Zoni II tanker oil spill on the marine ecosystem of Saronikos Gulf

**DOI:** 10.1016/j.dib.2019.104664

**Published:** 2019-10-16

**Authors:** Constantine Parinos, Ioannis Hatzianestis, Styliani Chourdaki, Elvira Plakidi, Alexandra Gogou

**Affiliations:** Institute of Oceanography, Hellenic Centre for Marine Research (H.C.M.R.), 46.7 Km Athens-Sounio av., Mavro Lithari, 19013, Anavyssos, Attiki, Greece

**Keywords:** Polycyclic aromatic hydrocarbons, Heavy fuel oil, Group IFO 380, Oil pollution

## Abstract

These data relate to the research article entitled “Imprint and short-term fate of the Agia Zoni II tanker oil spill on the marine ecosystem of Saronikos Gulf” by Parinos et al., 2019 [1]. The dataset includes the concentrations of 32 individual compounds/groups of polycyclic aromatic hydrocarbons (PAHs) determined in 235 seawater samples and 55 sediment samples analyzed during the monitoring survey conducted by the Hellenic Centre for Marine Research (H.C.M.R.) following the September 2017 Agia Zoni II heavy fuel oil (HFO) spill incident in Saronikos Gulf, Greece. The survey effort included 69 seawater sampling sites, of which 55 coastal and 14 open sea areas, and 22 sediment sampling sites across the inner Saronikos Gulf, aiming to assess the spatial and temporal imprint of the spilled oil during the first six months from the incident. The data were acquired by means of gas chromatography - mass spectrometry, following proper pre-treatment of the collected samples. This dataset is, to the best of our knowledge, the very first PAHs record related to the Agia Zoni II oil spill incident, that should be of interest for future scientific research on this and HFO spills in general.

**Table:** Specifications Table

Subject	Environmental Science
Specific subject area	Oil spill pollution environmental impact assessment
Type of data	Tables
How data were acquired	Analytical instrumentation: Agilent 7890 GC equipped with an HP-5MS capillary column (30 m × 0.25 mm i.d. × 0.25 μm phase film) coupled to an Agilent 5975C MSD.
Data format	Raw
Parameters for data collection	235 seawater samples and 55 sediment samples were collected across the inner Saronikos Gulf, Greece, following the September 2017 Agia Zoni II oil spill incident. The survey network included 69 seawater sampling sites, of which 55 coastal and 14 open sea areas, and 22 sediment sampling sites, aiming to assess the spatial and temporal imprint of the spilled oil during the first six months from the incident.
Description of data collection	The collected seawater and sediment samples were treated following an analytical scheme comprising initially of a solvent extraction step and subsequently clean-up by silica-column chromatography. The final determination and quantification of the targeted 32 compounds/groups of polycyclic aromatic hydrocarbons was performed by means of gas chromatography – mass spectrometry.
Data source location	Saronikos Gulf, Greece. Seawater and sediment sampling sites are presented in the supplementary interactive map. Detailed coordinates are provided in the Supplementary Excel Worksheets 1-3 (Tables 1–3).
Data accessibility	With the article
Related research article	The data are submitted as a companion paper to the research article of Parinos et al., 2019 entitled “Imprint and short-term fate of the Agia Zoni II tanker oil spill on the marine ecosystem of Saronikos Gulf” published in Science of the Total Environment, 693, article no. 133568 [1]. https://doi.org/10.1016/j.scitotenv.2019.07.374

**Table:** 

**Value of the Data** • This dataset is the very first record of polycyclic aromatic hydrocarbons related to the Agia Zoni II oil spill incident. • This dataset provides concentrations of a wide range of polycyclic aromatic hydrocarbons in environmental samples acquired by means of gas chromatography – mass spectrometry. • The data included in this paper can be used as reference data for future scientific research on the Agia Zoni II oil spill incident. • The data included in this paper can be used in future heavy fuel oil spill environmental impact assessment surveys in general.

## 1 Data

The data from the determination of the considered PAH compounds in the analyzed seawater samples collected from the coastal zone and open sea sites of Saronikos Gulf are provided in the Supplementary Excel Worksheet 1 (Table 1) and the Supplementary Excel Worksheet 2 (Table 2) respectively. The data from the determination of the considered PAH compounds in the analyzed sediment samples collected from open sea sites of Saronikos Gulf are provided in the Supplementary Excel Worksheet 3 (Table 3).

## 2 Experimental design, materials, and methods

Seawater samplings at various sites of the coastal zone of Saronikos Gulf were conducted over 10 sampling campaigns from September 18th 2017 to March 21st 2018 (55 sampling sites; 167 seawater samples in total, see Table 1). The water samples (2.5 L volume) were collected by means of a sampling device consisting of a weighted bottle holder with a clean amber-glass bottle and Teflon-lined cap [2]. Moreover, seawater samplings were undertaken on September 21–22nd 2017 and November 13–14th 2017 in open sea sites of Saronikos Gulf from the sea surface and various water depths of the water column (14 sampling sites; 68 seawater samples in total, see Table 2). In this case seawater samples were collected with 10 L Niskin bottles mounted on a rosette sampler and subsequently 2.5 L of seawater were immediately transferred to clean amber-glass bottles with Teflon-lined caps. All collected seawater samples were not filtrated, each one of them was preserved with 50 mL of Suprasolv *n*-hexane and were immediately transferred back to the laboratory where were analyzed within two to three days of collection. Sediment samplings in open sea sites of Saronikos Gulf were conducted on September 21–22nd 2017, November 13–14th 2017 and January 23–24th 2018 (22 sampling sites; 55 sediment samples in total, see Table 3). All sediments (top 1-cm) were collected using a stainless steel Box Corer with a surface area of 40 × 40cm and were wrapped in pre-combusted aluminum foil and immediately stored at −20 °C till further analysis.

In the collected seawater and sediment samples 32 individual compounds/groups of PAHs were determined, including the parent compounds with molecular weights ranging from 128 to 278, and the methylated derivatives of naphthalene, dibenzothiophene, phenanthrene, pyrene and chrysene. The data were acquired by means of gas chromatography - mass spectrometry on an Agilent 7890 GC, equipped with an HP-5MS capillary column (30 m × 0.25 mm i.d. × 0.25 μm phase film), coupled to an Agilent 5975C MSD, following proper pre-treatment of the collected samples as described elsewhere [1,[3], [4], [5], [6]].

The accuracy of PAHs determination in seawater samples, evaluated in terms of repeatability of the experimental results (*n* = 7; in spiked seawater samples) and expressed in terms of relative standard deviation ranged from 1.8 to 5.1% for individual PAHs. Detection limit was 20 pg L^−1^ for all compounds and the % expanded uncertainty (95%, k = 2) for individual PAHs ranged between 7.2 and 13.7%. The accuracy of PAHs determination in sediment samples was evaluated by analyzing the National Institute of Standards reference sediment SRM 1941b - NIST USA (organics in marine sediment). The determined values ranged between 92.4 and 108.3% of the certified values. The precision in the analysis of the samples, evaluated in terms of repeatability of the experimental results (*n* = 7) and expressed in terms of relative standard deviation, ranged from 1.4 to 5.9% for individual PAHs. Detection limit ranged from 0.05 to 0.26 ng g^−1^ for individual PAHs and the % expanded uncertainty (95%, k = 2) between 15.1 and 37%. The organic chemistry laboratory of H.C.M.R. is accredited by ISO/IEC 17025 for the analysis of PAHs in marine waters and sediments.
